# Patterns of Stochastic Behavior in Dynamically Unstable High-Dimensional Biochemical Networks

**DOI:** 10.4137/grsb.s2078

**Published:** 2009-01-29

**Authors:** Simon Rosenfeld

**Affiliations:** National Cancer Institute, EPN 3108, 6130 Executive Blvd, Rockville, MD, 20892

**Keywords:** nonlinear dynamics, stochasticity, gene expression, asymptotic stability, biochemical networks

## Abstract

The question of dynamical stability and stochastic behavior of large biochemical networks is discussed. It is argued that stringent conditions of asymptotic stability have very little chance to materialize in a multidimensional system described by the differential equations of chemical kinetics. The reason is that the criteria of asymptotic stability (Routh-Hurwitz, Lyapunov criteria, Feinberg’s Deficiency Zero theorem) would impose the limitations of very high algebraic order on the kinetic rates and stoichiometric coefficients, and there are no natural laws that would guarantee their unconditional validity. Highly nonlinear, dynamically unstable systems, however, are not necessarily doomed to collapse, as a simple Jacobian analysis would suggest. It is possible that their dynamics may assume the form of pseudo-random fluctuations quite similar to a shot noise, and, therefore, their behavior may be described in terms of Langevin and Fokker-Plank equations. We have shown by simulation that the resulting pseudo-stochastic processes obey the heavy-tailed Generalized Pareto Distribution with temporal sequence of pulses forming the set of constituent-specific Poisson processes. Being applied to intracellular dynamics, these properties are naturally associated with burstiness, a well documented phenomenon in the biology of gene expression.

## Introduction

“Will a large complex system be stable?” This question posed by R. May in his seminal paper^1^ in 1972 has reverberated in many hundreds of papers since then. In qualitative terms, the question may be reformulated as follows: Is that possible that a large collection of units interacting at random would create a stable system? We write this paper with intracellular biochemical networks in mind, such as those involved in gene expression (see^2^ for a more detailed discussion). It is obvious, however, that the same question is equally applicable to many other types of systems, such as predator-prey food chains, socio-economic structures, language, termite colonies, internet, energy and traffic infrastructures, to name just a few.

The properties of being large and being complex require some deliberation. Obviously, a system may be called large if it consists of a large number of individual elements. An ideal gas in a vessel of a macroscopic size is *a large* system because it contains 6·10^23^ molecules per mole. This system, however, cannot be regarded as *complex* since all the elements interact by simple laws of classical or quantum mechanics that are uniformly applicable to all the events of interaction. One may call a system complex either if there is a wide variety of interactions between the system’s components, or if the system consists of a large number of distinctly different subsystems interacting with each other, or both. Thus, one may call transportation infrastructure in a big city to be a large, but not complex, system, whereas the city itself is a large and complex system. It is typical for such systems that the disturbances that occur in one layer of the system easily penetrate to other layers threatening its entire collapse if not taken care of. Due to numerous connotations which the term *complex* may have in various contexts, in this paper we will refer to a complex system as *diverse*.

There are four major mechanisms of maintaining stability in large diverse systems. First, the system may be controlled by some overlaying structure which is different from the system-to-be-controlled and largely independent on it. Continuing the analogy with a transportation infrastructure, a minor car accident causing a traffic jam in a city can lead to a large-scale citywide disaster due to the domino effect propagating to all the levels of the system. In reality, however, such unfortunate developments are rare events despite numerous daily car accidents, and a natural question to ponder about is why it is so? The reason is that the transportation system *per se* is externally *supervised*. That is, there are other overlaying systems that have information-gathering capabilities, energy resources and independent transportation tools (e.g. police, helicopters, traffic cams, etc.) always ready to intervene into any unwanted event to prevent irreversible consequences. It is also important to realize that any such *supervision* always involves a “mind” of some sort, either genuinely human or the one mediated through large-scale computerized systems. Therefore, in a very general sense, an actual stabilizing force of supervision is in *understanding* the processes in the system-under-control and awareness regarding the ways of suppressing instabilities which may occur naturally and spontaneously.

Another scenario of stability in a large and diverse system is the case where a system is *stable-by-design*. To avoid misconception, we need to note that the word *design* is taken here in its literal meaning, which is, according to the *Cambridge Dictionary of American English*, “an outline, sketch, or plan, as of the form and structure of a work of art, an edifice, or a machine to be executed or constructed”. A similar definition is given in the *Encyclopaedia Britannica*. The concept of *stability-by-design* means that there exists a *designer* who intentionally provides a system with a set of hard-wired feedback loops for suppressing instabilities should they occur. Design and building of such a system is usually a cumbersome technical task involving sophisticated mathematical modeling, extensive testing, large-scale optimization and multifunctional decision making. Knowledge of laws governing the system is again a key element in creating a system stable-by-design. In evolutionary terms, only the *fittest* system has a chance to survive, but it is important that the *selection* in such a process should be regarded as *unnatural.* This means that it occurs not due to some kind of natural competition between the systems, but rather, it is the designer’s mind that decides which device is the fittest. In qualitative terms, the result by R. May cited above basically states that if we provide all the necessary components and allow them to interact randomly, i.e. to be driven only by blind forces without a designer, then the probability that sooner or later a stable configuration will be self-assembled is infinitesimally small in a large diverse system. Notably, examples in^1^ are taken from the predator-prey population dynamics and assume that the system may be regarded as large when the number of subunits is in dozens. It is a far cry from the genetic regulatory systems in which dimension may easily surpass thousands.

Due to the vast complexity of biological systems, and due to the unavoidable limitations of common language in characterizing their numerous properties and behaviors, it often happens that the terminology developed in a certain domain of human experience percolates into biology bearing only superficial similarity. In this context, it is sometimes speculated that in biology the role of designer may be ascribed to evolution; to a certain extent this may create no conflicts in understanding. However, in the context of this paper, we insist that the notion of *stability-by-design* should be strictly segregated from the notion of *stability-by-evolution*. This is because evolution, in the Darwinian sense, is thought to be a blind process of trial and error; it unfolds without the participation of a supervising mind which could be attributed to *a designer*. Although the term *design principles* is often applied to naturally occurring biological phenomena, strictly speaking, it is a misnomer; a *design principle* in a biological contexts reflects a natural order of things rather than a plot envisioned and implemented by an intelligent *designer*. It is especially important for a natural scientist to keep this terminology unambiguous and avoid verbal similarities with the theories of *intelligent design*. At last, the concept of *dynamical stability* should be strictly distinguished from the concept of *biological robustness*. Dynamical stability, as we are discussing it in this paper, is thought of as a property of the *dynamical systems* in their narrow mathematical sense, that is, systems that can be described by deterministic time-dependent differential and/or difference equations. Below, in the Discussion section, we offer some further reflections on these important topics.

Both of the above scenarios of stability, i.e. stability under supervision and stability-by-design, involve external control by some kind of intelligent being who uses its/his/her understanding of the laws governing the system for designing it to be stable or/and for suppressing spontaneous instabilities. The third possibility for the system to be stable involves the concepts of dynamical equilibrium and asymptotic stability. A fundamental difference with the first two possibilities is that, generally speaking, a dynamical equilibrium may occur by itself as a natural consequence of the laws governing the system. This scenario of stability is a central topic of this paper and will be addressed in more detail in the next section. Here we only mention that the conditions of equilibrium and asymptotic stability are tremendously complex in multidimensional systems of any nature, including high-dimensional biochemical networks. It is therefore important to understand what are the natural laws that guarantee validity of such complex rules of interaction in very large and diverse systems. Stated differently, since this kind of system is imbedded in any living cell, it is of great importance to understand what are the governing principles that bring such complex systems to stability and maintain this stability through generations of the cell. It is commonplace in biology to simply declare that such complex systems are the results of slow development under the so-called *evolutionary pressure*, which is a metaphor for the Darwinian principle of natural selection. By logical meaning, the notion of evolutionary pressure is nothing more than *a post hoc* justification of the existence of stable systems by the assumption that throughout the previous developmental history *there was* some natural purpose for that. Although it is not the author’s intention to dismiss the existence of the evolutionary pressure in principle, it should be nevertheless unambiguously stated that the mechanism of how this pressure actually works *on molecular level* is a wide open question still waiting to be resolved.^3^,^4^

The fourth guiding principle frequently invoked to explain stability of large systems is the *principle of self-organization*. The idea of self-organization stems from some fundamental facts established in theoretical physics. It has been shown in non-equilibrium thermodynamics that a system may be dynamically stable even far from thermodynamic equilibrium. In this case, a system may assume the form of the so-called *dissipative structure* in which dynamical stability is maintained at the expense of constant flow of energy through the system.^5^ There are a large number of experimentally observed phenomena—with Belousov-Zhabotinski auto-catalitic oscillations and Reileigh-Benard cellular convection being the celebrated examples—which are in agreement with this guiding principle.^6^ However, it is important to keep in mind that theoretical results, as well as experimental observations, available in the literature so far have never dealt with massively large and diverse systems. Quite the contrary, general theory usually encounters insurmountable mathematical difficulties in dealing with even low-dimensional systems. So far, the theory is able to elucidate self-organization (“pattern formation” is a more modern and, notably, more down-to-earth term^7^) only within extremely simple, largely abstract *toy models*. In experiments, even very simple systems in a stringently controlled environment still require careful handling and adjustments to be actually driven into a self-organized mode. Therefore, it is yet to be demonstrated that such small-scale manifestations of self-organization are indeed capable of serving as building blocks for large diverse stable systems.

The goal of this paper is to demonstrate that extremely stringent conditions of dynamical stability have very little chance to materialize in the realm of large biochemical networks. *We claim that any large biochemical network almost for sure is dynamically unstable.* It does not mean, however, that such a system is doomed to collapse due to implosion or explosion, as a simple linear analysis would suggest. It is possible that the system maintains a mode of existence in which the events of instability occur in a more or less random order, but generally, over a period of time, compensate each other.

The question of stability is of primary importance in studying genetic regulatory networks (GRN). The analogy with traffic in a big city discussed above is fully relevant to GRN. Dense interconnectedness of GRN is the reason why smooth behavior of GRN as a whole may be strongly dependent on seamless functioning of each gene. Proteins translated from mRNAs of some genes serve as transcription factors for many other genes; therefore, large sections of gene expression machinery may be halted by mere shortage, even temporary, of a few proteins that resulted from the transcription of other genes. Since the processes of protein production and delivery to appropriate regulatory sites are essentially random and involve many fluctuations and uncertainties,^8^ the *traffic jams* in such a system are rather mundane events. Since we assume (at least in the mainstream science, see^9^) that there is no supervisory intelligent system in the cell which *knows* where the bottleneck has occurred and which has independent resources to eliminate it, each such event may cause development of an avalanche of secondary events threatening to bring the entire system to collapse. This unavoidable sporadicity in GRN functioning has strong implications for gene expression profiling using modern high-throughput technologies.^10^ For instance, in microarray experiments, due to sporadic fluctuations of mRNA levels known as *burstiness*,^11^,^12^ the results of their measurements may be strongly dependent on the relation between duration of the mRNA harvesting and characteristic times between mRNA pulses. Therefore, a simplistic vision of mRNA profile as a more or less invariable attribute of the cell may lead to misinterpretations and errors. Poor reproducibility is an inevitable consequence of such an internal sporadicity and is a major obstacle for applications of microarrays in clinical practice.^13^

## Stability of Large Biochemical Networks: Deficiency Zero Theorem

Biochemical networks may be seen as a special case of nonlinear dynamical systems. As such, their stability may be studied by general principles of nonlinear dynamics. As discussed in,^2^,^14^ the Routh-Hurwitz and Lyapunov criteria of stability would impose a set of highly stringent constraints of high algebraic order on kinetic rates and stoichiometric coefficients. There are no known first principles or fundamental laws in statistical mechanics, thermodynamics and nonlinear dynamics that would draw a large diverse biochemical system into the state in which these constraints would emerge naturally, unless the system is externally controlled or/and is stable-by-design.

The Deficiency Zero Theorem (DZT) in *weakly reversible* chemical networks^15^,^16^ provides an additional avenue for studying stability, the one that does not have an analogue in the nonlinear dynamics in general. This theorem, a cornerstone of the *Chemical Reaction Network Theory*,^15^ is a far reaching generalization of the principle of detailed balance (PDB) known in classical chemical kinetics. In somewhat loose terms, the DZT states that weakly reversible chemical networks, i.e. the ones in which each direct chemical reaction is balanced by the *chain of inverse reactions*, are *globally asymptotically stable*, provided that the network has the deficiency zero. The *deficiency* of a network is an integer quantity, Δ = *m* − *l* − *s*, where *m* is the number of complexes, *l* is the number of linkage classes, and *s* is the rank of stoichiometric space (see^16^ for further definitions, details and extensive bibliography). It is also known that if a network *is not weakly reversible*, and/or if its deficiency *is not exactly zero*, then such a network is unstable. The DZT is a powerful statement which may serve as a solid heuristic principle in *designing* the stable reactors in chemical engineering. It is hard to escape a temptation to declare the DZT to be *a design principle of nature itself* and to hypothesize that the *evolutionary pressure* takes care about survival of only the systems which satisfy the DZT. Such a hypothesis, although highly attractive, would be a far reaching extrapolation of the facts at hand to completely unknown territories. It would also task the theory with a new fundamental problem of finding a natural (i.e. *unsupervised*) mechanism that draws a system into the state in which DZT is valid. We prefer to be cautious and side with J. Gunawardena^17^ saying that “It is still unclear to what extent the Chemical Reaction Network Theory is directly applicable to biological settings. Even if it is not, by understanding its mathematical basis we might hope to derive other results that are more appropriate to biology.”

In this work, we intend to go beyond a mere expression of doubt and to conjecture that in the vast majority of intracellular biochemical networks, the DZT *cannot be valid*. There are at least two reasons for such a conjecture. First, in a system where the number of chemical species is in hundreds of thousands and the number of chemical reactions between them perhaps in millions, it is difficult to envision how the deficiency zero can be maintained. Even if such a precise balance does exist at a certain stage of the lifecycle of a cell, random partitioning during mitosis would immediately destroy it.^18^ Numerous processes of intrinsic stochasticity in gene expression (often disrespectfully called simply *noise*!^19^) make it impossible to even characterize the system in terms of ordinary differential equations, as required by DZT; stochastic differential equations are widely viewed to be a more appropriate analytical tool.

Second, in biochemical networks even the very notion of *a system* is largely uncertain and admits wide latitude in selection of compartmentalization, descriptors and quantitative characterizations. For example, on a certain level of abstraction, the process of transcription may be seen as an individual biochemical reaction between RNA polymerase and DNA molecule, whereas a more detailed view reveals a complex sequence of rearrangements involving hundreds of molecules and thousands of elemental steps, each representing a separate chemical reaction.^20^ Obviously, the fact of presence or absence of global stability cannot depend on the granularity of details a researcher selects for modeling the system. Therefore, in order for the system to be stable, the deficiency zero requirement has to be, so to speak, *structure-invariant*. This is a draconian requirement! It is hard to believe that real-life systems indeed have natural mechanisms to maintain such a structure-invariant deficiency zero. The global structure-invariant stability is only possible in the state of full thermodynamic equilibrium, and the PDB is a precise expression for that. The PDB, however, is not applicable to the *weakly reversible* chemical networks, the ones for which the DZT has been established.

## Qualitative Behavior of Large, Diverse, Inherently Unstable Biochemical Networks

Based on the analysis in the previous section, we come to the conclusion that in large diverse biochemical networks, the conditions of asymptotic stability are so stringent that they have very little chance to materialize, unless they are controlled externally or designed artificially. Hence, a natural question arises how do such unstable systems behave? This topic has been discussed in detail in the works^2^,^14^ by the author. In this paper we provide a brief summary.

A natural basis for the description of chemical kinetics in a multidimensional network is the power-law formalism, also known as S-systems. Being algebraically similar to the Law of Mass Action (LMA), S-systems proved to be a useful tool in the analysis of complex biochemical systems and metabolic pathways. Importantly, in the vicinity of equilibrium *any* nonlinear dynamical system may be represented as an S-system.^21^ Unlike mere linearization which replaces a nonlinear system by the topologically isomorphic linear one, the S-approximation still retains essential traits of nonlinearity but often is much easier to analyze.

Let *F*(*x*) and *G*(*x*) be the vector-functions, *R*_+_*^N^* → *R*_+_*^N^*. We consider an autonomous dynamical system

(1) dx/dt=F(Px)-G(Qx),

where *P* & *Q* are matrices of all positive elements such that *P*−*Q* is invertible. As shown in,^19^ dynamical system (1) always has at least one fixed point, *x*^0^, and in its vicinity the system is representable as

(2) $$\frac{{dx}_{i}}{dt}=\Phi_{i}(t|x_{0})=\alpha_{i}\text{exp}\left[\sum_{k}\xi_{i}P_{ik}x_{k}\right]-\beta_{i}\text{exp}\left[\sum_{k}\eta_{i}Q_{ik}x_{k}\right]$$

with

$$\begin{array}{l} \xi =\nabla_{U}F;\eta =\nabla_{V}G;\alpha =\text{exp}\left[F(U^{0})-\xi U^{0}\right];\\ \beta =\text{exp}\left[G(V^{0})-\eta V^{0}\right],U^{0}={Px}^{0};V^{0}={Qx}^{0} \end{array}$$

Formally, system (2) is equivalent to an S-System^22^,^23^ describing the biochemical reactions with “concentrations”, exp(*x_k_*). In the vicinity of the fixed point, *x*^0^, the Jacobian matrix of (2) is neither symmetric nor anti-symmetric; hence, generally, its eigenvalues are complex numbers with both negative and *positive* real parts. The latter means that such systems are dynamically unstable. This conclusion is in line with the analysis by R. May.^1^

A conceivable scenario of behavior of such systems has been proposed by this author in^2^ and termed the *Stochastic Cooperativity Paradigm* (SCP). In the SCP we take into consideration the fact that in a system of an asymptotically large dimension, the vectors *Px* &*Qx* fluctuate around zero most of the time except the comparatively rare events of *stochastic cooperativity* when majority of the *x_k_*(*t*) simultaneously reach their respective maxima thus producing large sporadic *excursions*. These excursions are grossly amplified by exponentiation and result in a signal quite similar to the *shot noise*, thus giving rise to the Langevin-type equations

(3) $$\frac{{dx}_{i}}{dt}=\frac{1}{\tau_{i}}\sum_{k=1}^{L_{i}}\mu_{ik}\delta (t-t_{ik})$$

where *t_ik_* are the moment of excursions (*burstings*) and *μ_ik_* are random amplitudes. Qualitatively, the SCP signifies transition from the purely deterministic description containing in (1) to the stochastic description in terms of random walks and Fokker-Plank equations.

In,^14^ we have provided a more rigorous quantitative approach supporting these heuristic considerations of SCP. In the very core, the problem may be reduced, after a series of algebraic transformations, to studying the set of stochastic processes, *z*,

(4) $$dz/dt=h_{\sigma }(t)=\text{exp}[\sigma x(t)]-\text{exp}[\sigma y(t)],$$

where *x*(*t*) & *y*(*t*) are the (approximately) Gaussian processes and *σ* is the parameter controlling *complexity* of the network. Exact analytical form of the distribution of *h_σ_*(*t*) is unknown. We have shown by simulation that this process may be accurately represented through the Generalized Pareto Distribution (GPD)

(5) $$\begin{array}{l} G_{\xi ,\beta }(x)=1-{\left(1+\xi x/\beta \right)}^{-1/\xi },\xi \neq 0;\\ G_{\xi ,\beta }(x)=\text{exp}(-x/\beta ),\xi =0 \end{array}$$

with the parameters, *ξ* and *β*, depending on *σ*. These dependencies are found to be

(6) $$\begin{array}{l} \xi (\sigma )=-0.376+0.745\sigma -0.088\sigma^{2};\\ \beta (\sigma )=0.392\;[\text{exp}(1.162\sigma )-\text{exp}(-2.753\sigma )]. \end{array}$$

The fact that the process *h_σ_*(*t*) can be represented by a heavy-tailed GPD means that a substantial amount of its spectral energy is contained in the *exceedances*, that is, in short sporadic pulses beyond certain predefined bounds. If, for example, *σ* = 1.5, then the process *h_σ_*(*t*) spends about 95% of time between the 2.5% and 97.5% quantiles. Nevertheless, the variance of the exceedances beyond this interval is overwhelmingly greater than that within (7698 and 183, respectively). On this basis, one may regard *h_σ_*(*t*) as a pulse process slightly distorted by a small background noise. If we ignore the noise, then equation (4) is reduced to the Langevin form (3), where *t_ik_* is the set of (constituent-specific) random point processes coinciding with the events of *bursting*. Theory of level-crossings predicts that these processes are asymptotically, *a/σ* → ∞, equivalent to the Poisson processes with the parameter, *ζ*,

(7) $$\zeta =(1/2\pi )(1/\tau_{0})\text{exp}\left\{-a^{2}/[2\sigma^{2}]\right\},$$

where *a* is the threshold of excursions, and *τ*_0_ and *σ*^2^ are the correlation radius and variance of the generating Gaussian process, respectively. We have shown by simulation that (7) is valid for the sequences, *t_ik_* in (3) even when *a/σ* is not so big, say, *a* = 1.35*σ*. It is also worth mentioning that the density of peaks per unit of time generated by the process (4) is close to that predicted from the asymptotic theory (e.g. 696 and 703, respectively, within the interval of length 10000. These examples indicate that equation (7) is applicable under much milder conditions than *a/σ* → ∞. We may reach, therefore, an overall conclusion that the *stochastic* phenomenon of burstiness is implicitly contained in the purely *deterministic* dynamical description (1).

The fact that in a system of asymptotically large dimension, deterministic dynamics may be reduced to the Langevin equation describing a pseudo-random walk is significant. In classical statistical physics, such a derivation is only possible if the system is stable, and there is a trivial reason for that: random excitation should be balanced by deterministic damping. This is the essence of the fundamental Fluctuation-Dissipation theorem stemming from the seminal works by Einstein, Langevin and Smolukhovski.^24^ The approach offered here illustrates that even in an inherently unstable system, such as a biochemical network of very high dimension with non-zero deficiency, some kind of equilibrium is still possible since the sporadic excursions impact both production and degradation, and in the long run may balance each other.

## Burstiness in Genetic Regulation

As mentioned above, burstiness is a well documented phenomenon in gene expression. This phenomenon is given considerable attention in the literature.^25^ Usually, burstiness is associated with some special circumstances surrounding gene expression, for instance, with very low concentration of macromolecules of a certain type leading to essential discreteness of the process and large relative fluctuations. What is apparently overlooked in existing theories is that in the systems of such tremendous complexity, it is simply impossible that every transcription factor would be delivered in a timely, “assembly-line” manner to any regulatory site of any of 25,000 genes. Since the proteins transcribed from some genes serve as regulatory factors in numerous others, even a minor and temporary delay in supply immediately suspends the next set of transcription events downstream on the metabolic pathway, thus threatening to halt big interdependent sections of the system. The situation is quite similar to a traffic jam in a city. The difference, however, is that there is no supervisory intelligent being in the cell which is permanently on the alert for correcting the unwanted situations; therefore, returning back to normal should somehow happen by itself. The concept of stochastic cooperativity helps to envision a possible scenario of such self-correcting. If some group of genes temporarily stops functioning, then a number of the proteins transcribed from other genes remain unclaimed and begin to accumulate in excessive quantities, thus engaging alternative pathways for circumventing the stumbling blocks. After all the transcription factors find their alternative ways to the corresponding regulatory sites, the RNA polymerase begins to move and synthesize the mRNA. Figuratively speaking, all the transcription factors should first come to cooperation through assembling the team supporting RNA polymerase functioning. The entire regulatory process, therefore, is a sequence of sporadic events analogous to the above described events of stochastic cooperativity. Exact order of these events, i.e. trajectories in the phase space, may vary from time to time and from cell to cell. As experimentally observed in,^12^ even in two daughter cells after mitosis the exact sequences of turning the genes on and off may be quite different. Nevertheless, the entire ensemble of the trajectories may be described in probabilistic terms using the FPE reflecting the deterministic evolution of the probability. Smooth evolution of this probability is what in biology is conceptualized as stability.

## Discussion

In physics, formalized theoretical models of complex physical phenomena are often called *toy models*. This terminology explicitly highlights that fact the model is not intended to be a comprehensive theory behind a phenomenon; rather, it attempts to provide a reasonably realistic description of certain *core elements* of this phenomenon. Obviously, it would be a fallacy to attack a toy model on the basis that it is unable to provide a realistic description of the phenomenon in its entirety, however complex it is. In biology, this contrast between the theoretical toy models and the structure of complex biological entities is even more drastic than in physics. The theoretical considerations offered in this paper do not intend to provide a comprehensive framework for description of cellular dynamics in its entirety. It only focuses on one fundamental property of biochemical networks, that is, on their unavoidable *dynamical* instability. In qualitative terms, *asymptotic dynamical stability* is the property of dynamical systems of having trajectories that with time approach a certain bounded domain in the system’s phase space and stay within that domain forever.^26^ This definition covers both stable fixed points and stable periodic orbits (i.e. limit cycles). It is useful to keep in mind that *asymptotic dynamical stability* is a fairly rare occurrence in the world of multidimensional nonlinear systems. Therefore, it should not be regarded as a big surprise that a multidimensional system of intracellular biochemical reactions is dynamically unstable unless a set of extremely complex conditions for its stability are satisfied.

Observed biological robustness of living organisms and their resilience in the face of external perturbations is not a counterargument to dynamical biochemical instability. Robustness differs from stability in that it deals with maintaining *the system’s functions* as opposed to *the system’s states*.^27^ Generally, biological robustness does not assume structural invariance of the system: the fail-safe mechanisms for maintaining homeostatic equilibrium may turn on and off as required by different circumstances.^28^ A tremendous asset in the struggle for functional stability is the modular structure of large biological systems;^29^ it prevents the system from global failure should such a failure occurs in an individual module. The question to be resolved by science is how functional robustness can originate from the elements that are inherently dynamically unstable. The seeming contradiction between functional stability of a vast organizational structure consisting of a large number of biochemical networks and possible dynamical instability in each of them is fictitious; it attempts to oppose different levels of biological organization. A logically satisfactory way of looking into these issues is through the paradigm called *dual causality* formulated by Palsson.^30^ He writes in Chapter 12: “Unlike physiochemical sciences, biology is subject to dual causality or dual causation. Biology is governed not only by the natural laws but also by genetic programs. Thus, while biological functions obey the natural laws, their functions are not predictable by the natural laws alone. Biological systems function and evolve under the confines of the natural laws according to basic biological principles, such as generation of diversity and natural selection. The natural laws can be described based on physicochemical principles and used to define constrain under which organisms must operate. How organisms operate under these constrains is a function of their evolutionary history and survival.” Within the paradigm of *dual causality*, inherent dynamical instability represents the “natural laws” and “physicochemical principles” whereas biological robustness is a result of evolutionary history in which this dynamical instability is either suppressed or is effectively used for gaining evolutionary advantages and survival. The role of fast fluctuations in evolution has been extensively discussed in the literature.^4^ In essence, what is shown in our paper is that such fluctuations in a system are not necessarily created by *external forces*, and do not necessarily originate from *unmodeled realities within* the system. They may be a natural consequence of high dimensionality coupled with high nonlinearity. The notion that short-term instabilities may manifest themselves as “noise” at a higher level of organization is not new and has a long history in nonlinear dynamics (see^31^ and references therein). It is also worth mentioning that dynamical instability may play an essential role in the very process of search for stability; without fluctuations created by these instabilities, a system would not be capable of exploring the topology landscape around its current state and making a step towards stability.^5^,^32^

The engineering concepts of negative and positive feedback regulatory loops have been extensively used in the analyses and interpretations of complex biological data (e.g.^33^,^34^). However, in biological literature, the prerequisites which make application of such concepts to *biochemical networks* justifiable are rarely formulated explicitly or even mentioned. Since biochemical networks, whether high- or low-dimensional, are strongly nonlinear dynamical systems, such prerequisites are very far from trivial. First of all, the system has to possess at least one asymptotically stable regime, either in the form of a fixed point or of a limit cycle. As seen from the analysis presented in the previous sections, a number of intricate criteria should be satisfied to make such asymptotic stability possible. If a stable asymptotic regime does exist, then the deviations from this regime may be analyzed (with an additional assumption of “smallness” of perturbations) using the concepts of linear theory. This step is equivalent to the Jacobian analysis of (linear) stability in which the eigenvalues with negative and positive real parts would correspond to negative and positive feedback loops, respectively. Only negative feedback loops provide stability, and the conditions for their existence (i.e. for negativity of real parts of eigenvalues) are very complex. In biological studies, all these important prerequisites are often either bypassed or *assumed to be in place* or simply *postulated* to be the natural outcomes of evolution. Quite a characteristic example may be found in a comprehensive treatise devoted to biological feedback by Thomas and D’Ari.^35^ After careful discussion of verbal, logical, and differential levels of description in biology (Introduction), and after stability analysis of logical cycles (Chapter 3), we find the following statement (page 65): “If we wish to find logical solutions in which our cycle is a stable attractor, the easiest way is to *impose* stability, rather than testing the stability of the cycle in randomly chosen combinations of functions” (italics by Thomas and D’Ari). In this example, as well as in innumerable other instances scattered throughout the biological literature, the conditions of stability are simply *imposed*. Metaphorically speaking, if it is assumed that elephants are capable of flying, then, guided by this assumption, a nice theory may be developed regarding mechanics and aerodynamics of their flight. Furthermore, the theory may become a basis for data analysis and model parameterization, and should a contradiction occur between the theory and observations, it may be attributed to the laws yet unknown and to be discovered in future. The essence of this metaphor is that as long as the question of stability of high-dimensional biochemical networks is not carefully addressed, all the results based on the *assumption* of stability remain purely phenomenological and therefore lacking a solid theoretical basis and predictive force.

The question of dynamical stability of large biochemical networks is not of a purely academic interest but has far reaching practical implications in the systems and computational biology. It is often the case that the software packages specifically designed for computational modeling of intracellular biochemistry leave the question of asymptotic stability largely unaddressed. These packages—usually equipped with easy-to-use graphical interfaces and convenient scripting languages—allow one to design any imaginable system in chemical kinetics (see,^36^ Table 1.3, and^37^). What is easy to overlook in this design is the question of dynamical stability. If no attempts are made to take care of asymptotic dynamical stability, then according to the central result by R. May,^1^ the probability that the system turns out to satisfy these conditions due to a miraculous coincidence is miniscule. Therefore, almost surely such a model will be dynamically unstable. As known from mathematics, if a system does not possess the property of asymptotic stability then, in a time-course dynamics, its computational convergence to a certain limit may have nothing to do with the properties of the system-to-be-modeled; it may be a purely computational artifact. To avoid such an unpleasant situation, existence of a stable steady state is often *hypothesized*, and only small perturbations to this hypothesized state are actually modeled. However, as follows from the above discussion, existence of such a steady state should not be taken for granted. It should be either proven mathematically or should have a very strong experimental justification. Heeding the lessons learned from biology, one may expect that *modularity* and careful *separation of time scales* would serve as a stabilizing measure in computational models. These questions are discussed in more detail in the recently published work^38^ by the author.

## Conclusion

The central message of this paper is extremely simple and may be easily expressed without explicit mathematical modeling. A large diverse network with the number of units in tens of thousands and link density in hundreds cannot behave in a smooth assembly-line manner. Spontaneous failures like traffic jams, bottlenecks, backlogs, delays, loss of synchronization, etc., are absolutely unavoidable circumstances surrounding their functioning. In the absence of independent external forces capable of supervising and quickly repairing these failures, each of them initiates a wave of secondary failures thus moving the system unidirectionally towards destabilization. In the systems where dynamics is a game of many conflicting forces, such as source versus sink, production versus degradation, attraction versus repulsion, this destabilization may impact both parts of the game. As in a comedy of errors, these opposite tendencies in the long run may compensate each other thus bringing the system to erratic, but generally successful, functioning.
